# Supplementary figures and images for: A juxtamembrane basolateral targeting motif regulates signaling through a TGF-β pathway receptor in Drosophila
Source: PLoS Biol. 2022 May 20;20(5):e3001660. doi: 10.1371/journal.pbio.3001660 (PMC9162340; doi:10.1371/journal.pbio.3001660)

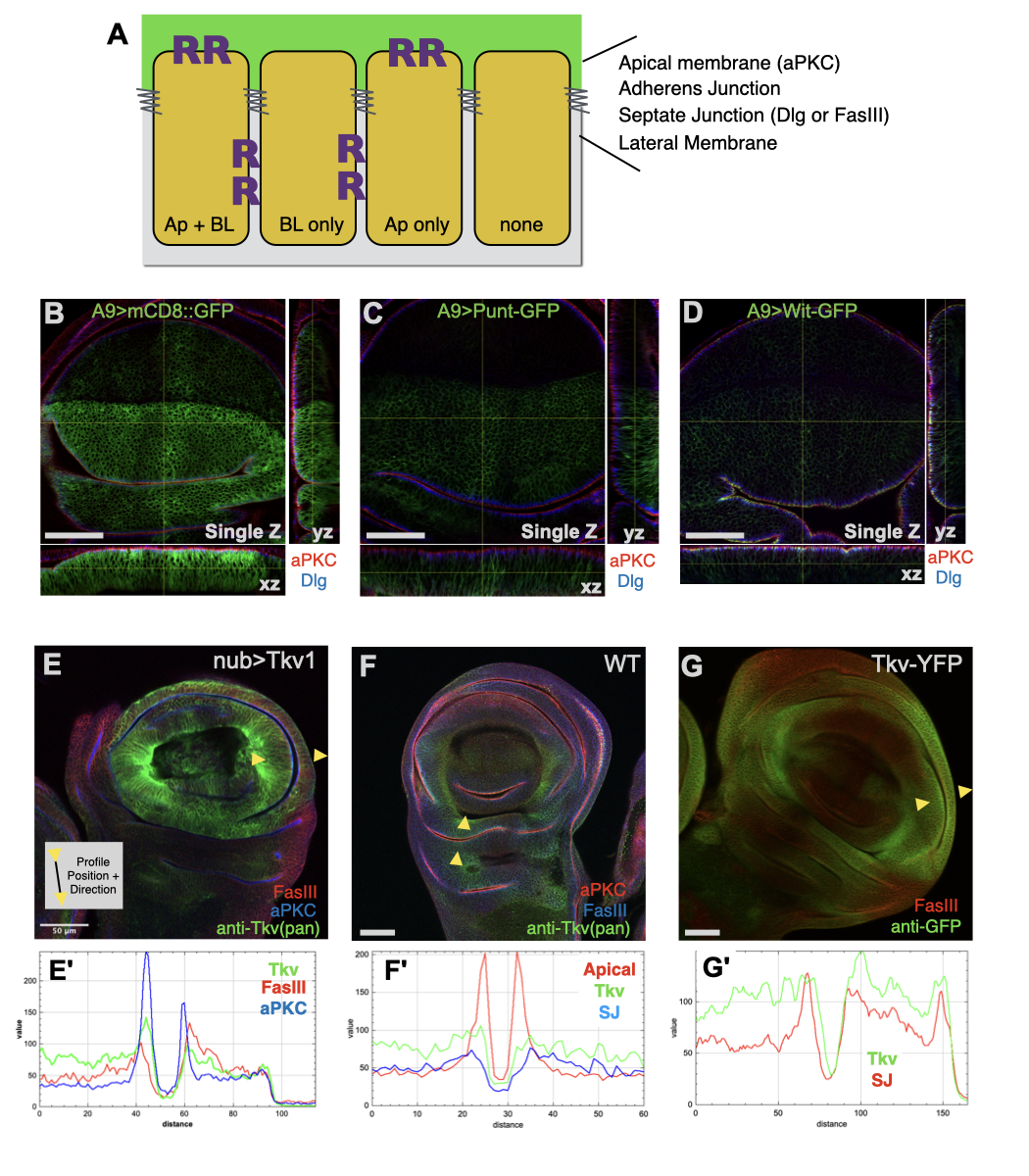

Supplement: S1 Fig — (A) Simplified model of an epithelium with 2 ligand compartments (green and gray) and 4 distribution patterns of a receptor R based on Ap or BL presentation. Markers for specific membrane regions used in this study [2]. (B–D) Confocal imaging of wing discs to reveal distribution of junctions and apicobasal regions. The continuous epithelium of the disc has characteristic folds that bring the apical sides of 2 regions facing toward each other, which appear as “wrinkles” or “folds” in a single confocal plane. (E–G) Localization of Tkv relative to membrane domains. Tkv1 isoform overexpressed in the wing disc detected by anti-Tkv(pan) and compared to FasIII SJ marker and aPKC apical marker (E), with profile (E′) recorded at position indicated by yellow arrowheads. Isoforms Tkv1 and Tkv2 from Brummel and colleagues [53] correspond to Tkv-D and Tkv-A isoforms, which differ at the N-terminus. Endogenous Tkv1 and Tkv2 are expressed at low levels in the wing so the IF signal is dominated by the overexpressed protein in the nub-GAL4 domain. Endogenous Tkv detected with anti-Tkv(pan) antibody also showed a general membrane distribution (F and F′). Similar results were obtained for anti-GFP staining of a tkv allele expressing Tkv-YFP (G and G′). Ap, apical; BL, basolateral; IF, immunofluorescence; SJ, septate junction. (TIFF) [file pbio.3001660.s001.tiff]

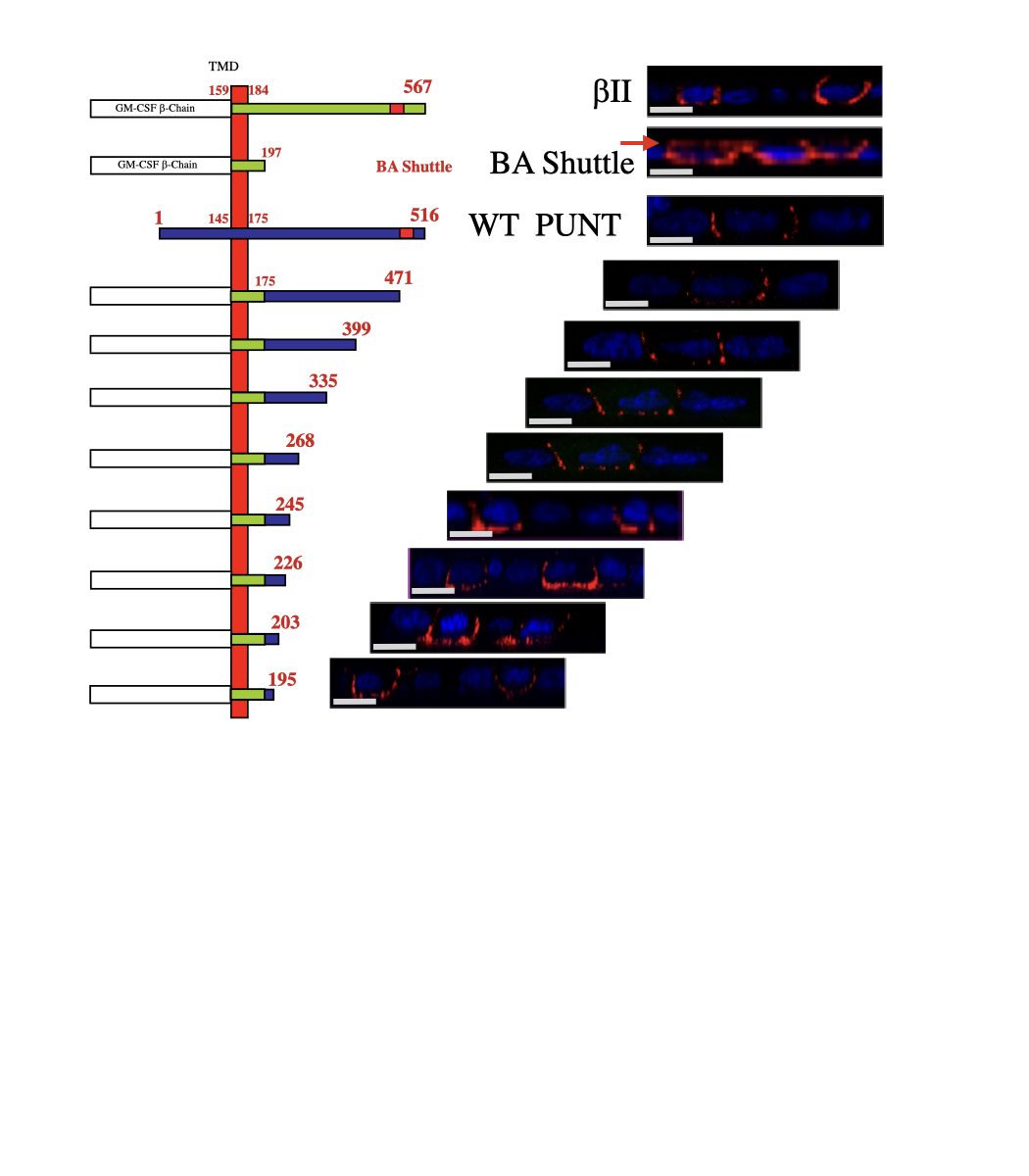

Supplement: S2 Fig — Schematic of the component segments of receptors tested for basolateral restriction is shown to the left, and a representative xz confocal projection to the right. In the diagram, the ectodomain is to the left of the membrane, which is marked by vertical red bar, and the cytoplasmic domain is to the right. In the images, apical is up and nuclei are stained in blue with DAPI. The βΙΙ protein from Murphy and colleagues displays basolateral localization of a chimeric receptor containing the TβRII cytoplasmic domain. The BA shuttle lacks the required LTA motif, depicted by the red rectangle within the green TβRII protein, and thus displays an unrestricted apicobasal disribution (red arrow points to apical staining). WT Punt is also restricted to the basolateral membrane domains, as are chimeric proteins containing various cytoplasmic portions of Punt. The corresponding LTA position, shown by the red rectangle in the blue Punt protein, is not required for the basolateral localization since progressive carboxyl-terminal truncations lacking this region retain the activity. The shortest truncation tested possessed only a short stretch of the cytoplasmic portion of Punt. There are di-leucine elements at positions 196+197 and 204+205, but neither is required for basolateral restriction since the shortest truncation ending at amino acid 195 retains basolateral localization. MDCK, Madin-Darby canine kidney; TβRII, TGF-β Type II receptor; WT, wild-type. (TIFF) [file pbio.3001660.s002.tiff]

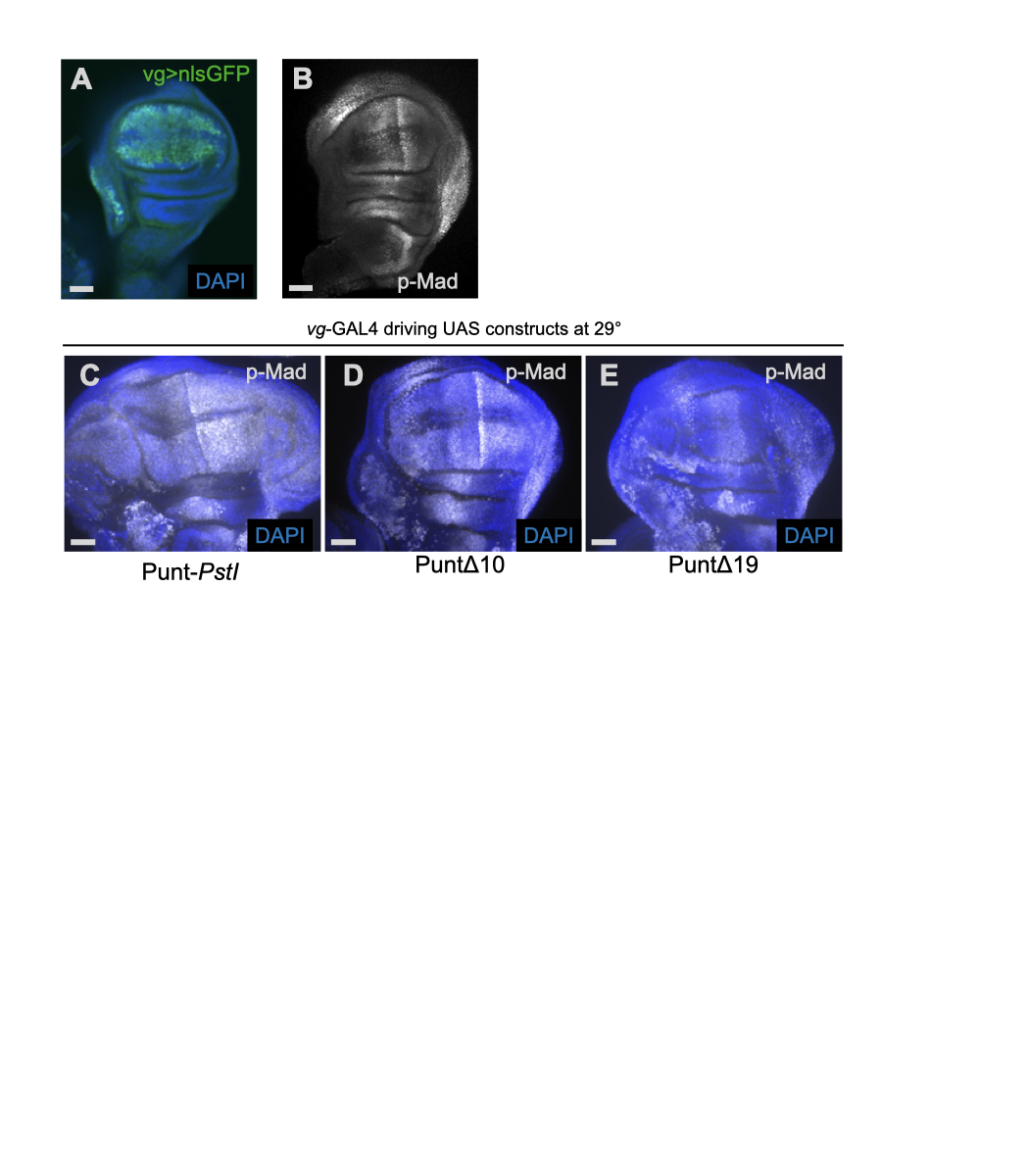

Supplement: S3 Fig — (A) vg-GAL4 expression pattern in third instar wing disc as shown by UAS-nlsGFP reporter (green; DAPI in blue), single confocal section. (B) p-Mad detection pattern in WT disc, maximum intensity projection. (C–E) p-Mad signal upon overexpression of intact Punt (C) and protein variants missing part (D) or all (E) of the BLT motif. p-Mad in white, DAPI in blue. Punt (Punt-PstI) and both deletions generate ectopic pMad in the pouch and at anterior side of disc. Widened discs are consistent with overgrowth caused by excess Dpp/BMP signaling (compare widths in C–E to control discs in A and B). Note that these constructs are inserted at random genomic positions with different expression levels, so the degree of ectopic signaling cannot be correlated with protein deletions in this context. Anterior is to the left in all images. Scale bars: 50 μm. BLT, basolateral targeting; WT, wild-type. (TIFF) [file pbio.3001660.s003.tiff]

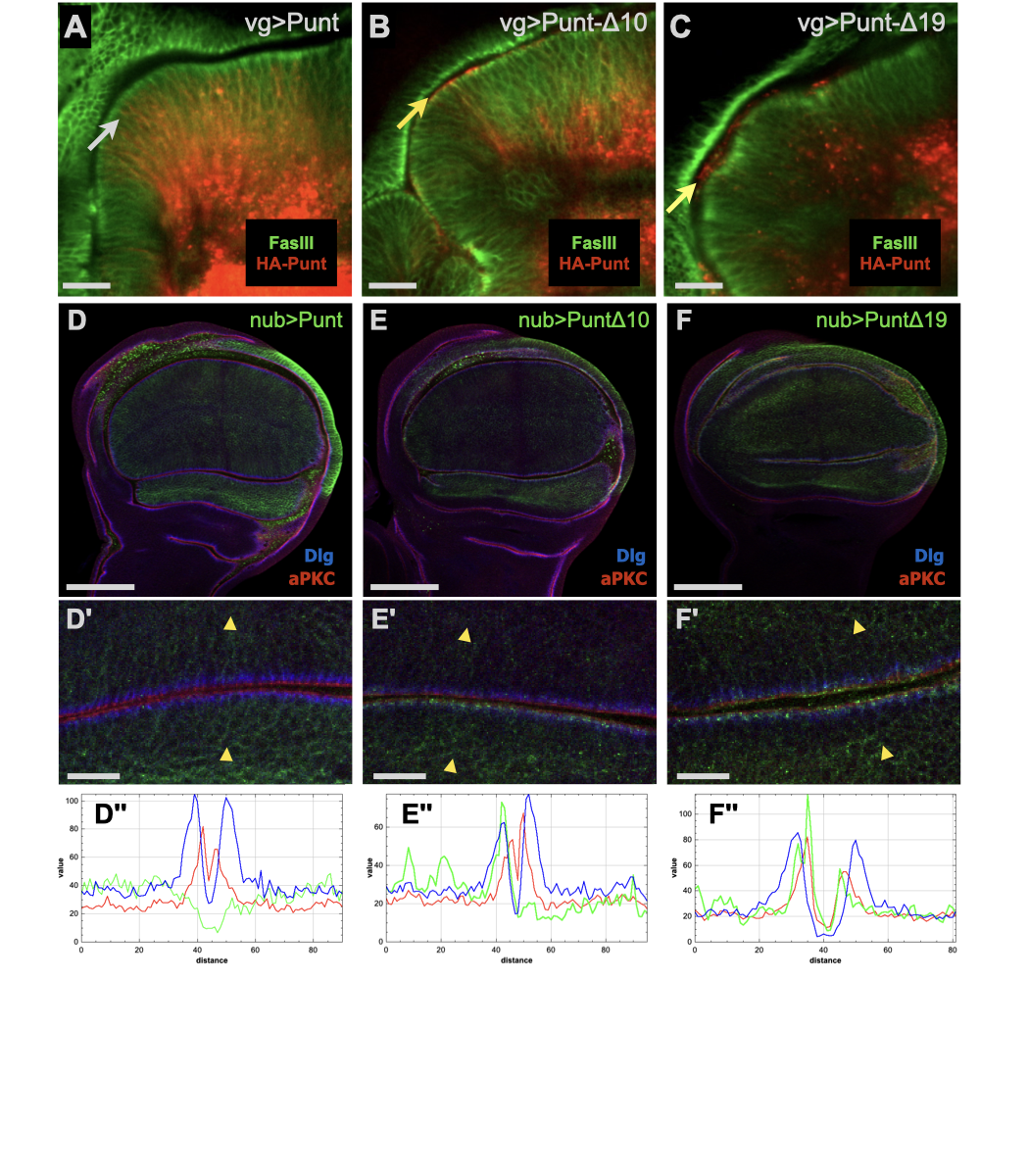

Supplement: S4 Fig — (A–C) Punt-HA variants expressed with vg-GAL4 detected with an antibody for the extracellular HA epitope. White arrow in A indicates extent of Punt detection signal, which remains basal to the junction. Yellow arrows in B and C indicate ectopic staining apical to the junctions. (D–F) Punt-HA variants expressed with nub-GAL4 detected with cytoplasmic anti-Punt antibody. Punt detection is absent at or apical to the SJs (D). PuntΔ10 shows significant overlap with the SJ but in many areas is excluded from the apical membrane (E). PuntΔ19 shows significant overlap with junction and apical regions (F). Single prime images are close-up views; yellow arrowheads indicate position and direction of profiles displayed in double prime panels. Scale bars: 50 μm A–C, 100 μm D–F, 15 μm D′–F′. BLT, basolateral targeting; SJ, septate junction. (TIFF) [file pbio.3001660.s004.tiff]

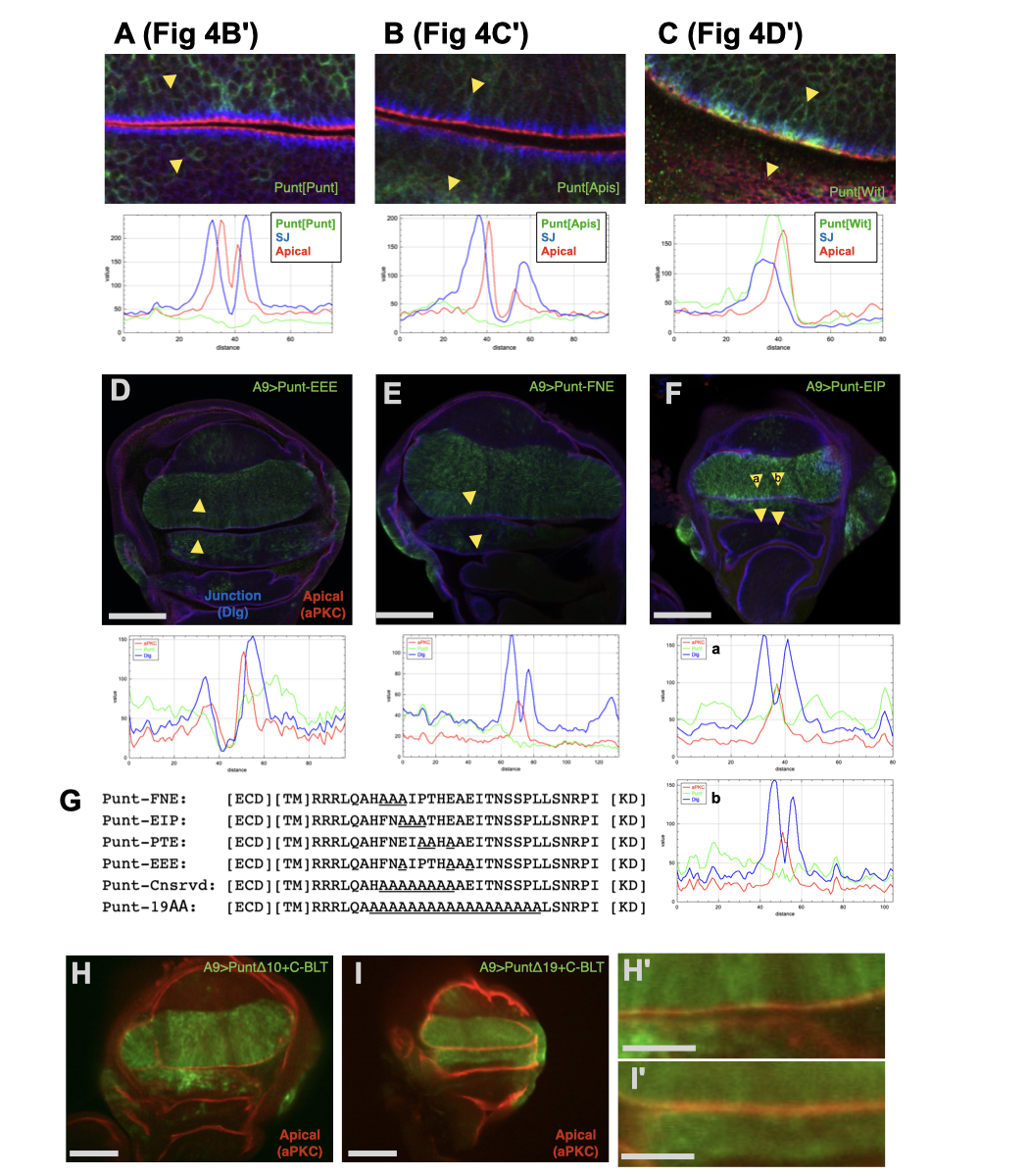

Supplement: S5 Fig — (A–C) RGB profiles for Punt BLT region substitution constructs, with corresponding Fig 4 panels indicated. The control Punt[Punt] and Punt[Apis] proteins are largely confined to the lateral membranes, and excluded from junction and apical membrane domains (A, B). Punt[Wit], a Punt protein harboring a stretch of Wit amino acids only in the juxtamembrane region, showed unrestricted distribution with an apical preference (C). (D–F) Localization of point mutant versions of Punt. The EEE, FNE, and EIP and triple point mutants generally retained basolateral presentation, but the EIP mutant had variable regions of apicalization (compare profile examples a and b below F). Direction and position of profile line are indicated by yellow arrowheads, as indicated in D inset. (G) Sequence of BLT point mutants; all include the K and L mutation from PstI cloning scar. (H, I) Addition of the BLT to carboxyl terminus of otherwise apicalized Punt proteins. L3 wing discs dissected and stained for Punt and apical aPKC. Constructs had either 10 or 19 residues from the BLT region deleted and the BLT appended to the carboxyl terminus. When the insect-conserved residues of the BLT region were deleted (Δ10) and the BLT was added carboxyl-terminally, apical mislocalization of Punt was still observed (H). Deletion of the entire BLT region (Δ19) and addition of the BLT carboxyl-terminally resulted in apical mislocalization of Punt, as seen by the yellow stripe of colocalization (I). H′ and I′ are enlarged to better show the apical stripe. Scale bars: 100 μm D–F, H, I; 50 μm H′, I′. BLT, basolateral targeting. (TIFF) [file pbio.3001660.s005.tiff]

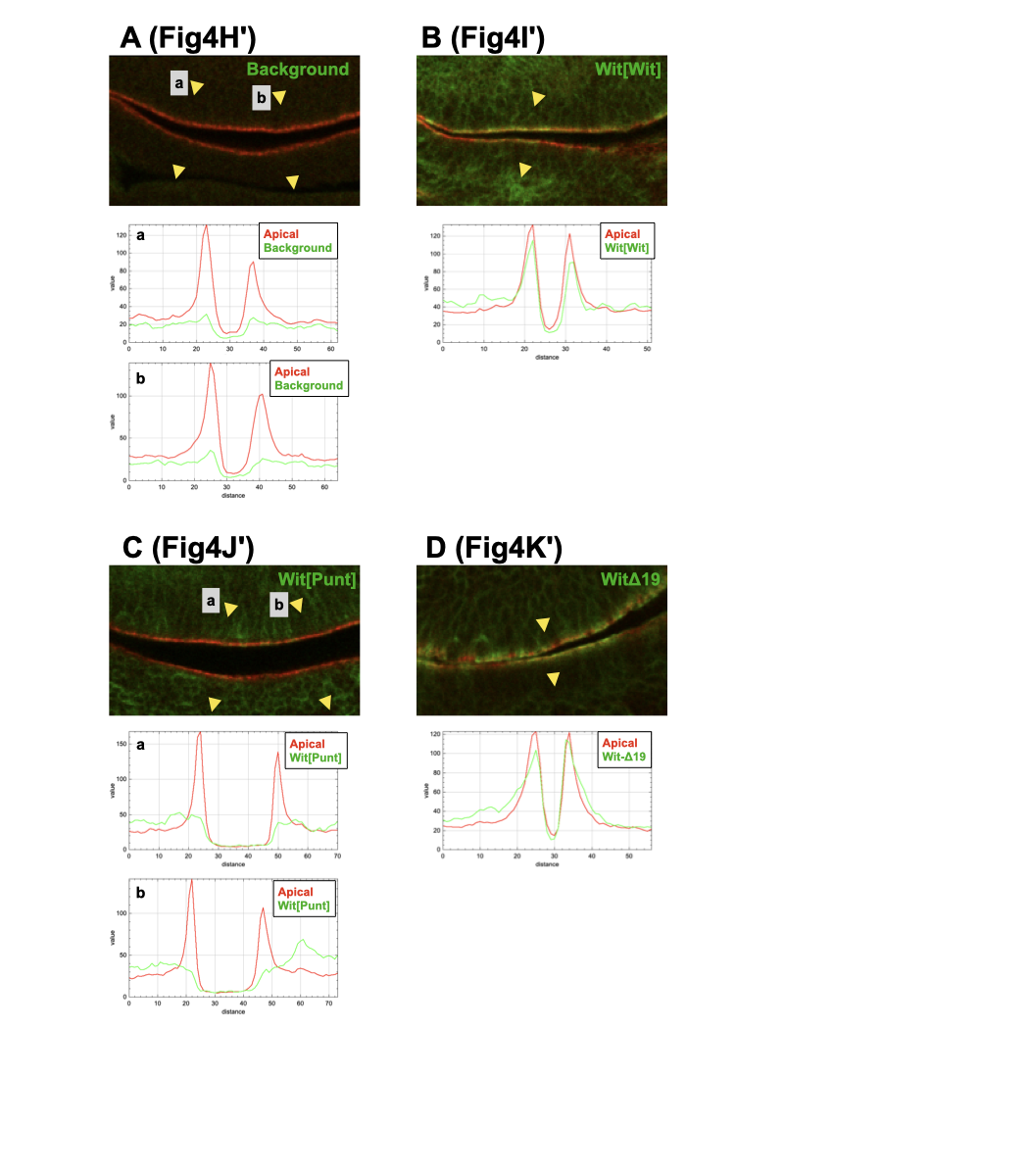

Supplement: S6 Fig — (A–D) Profiles of Wit proteins shown in Fig 4. Anti-FLAG IF in the wing disc displayed a significant background signal, with enrichment at the apical membrane (A). Wit[Wit] was detected in all regions, with an enrichment overlapping the apical membrane (B). Wit[Punt], which is a Wit protein with Punt amino acids only in the juxtamembrane region, had a localization more like Punt than Wit (C). Deletion of the Wit juxtamembrane region did not alter the protein’s apicobasal distribution (D). (TIFF) [file pbio.3001660.s006.tiff]

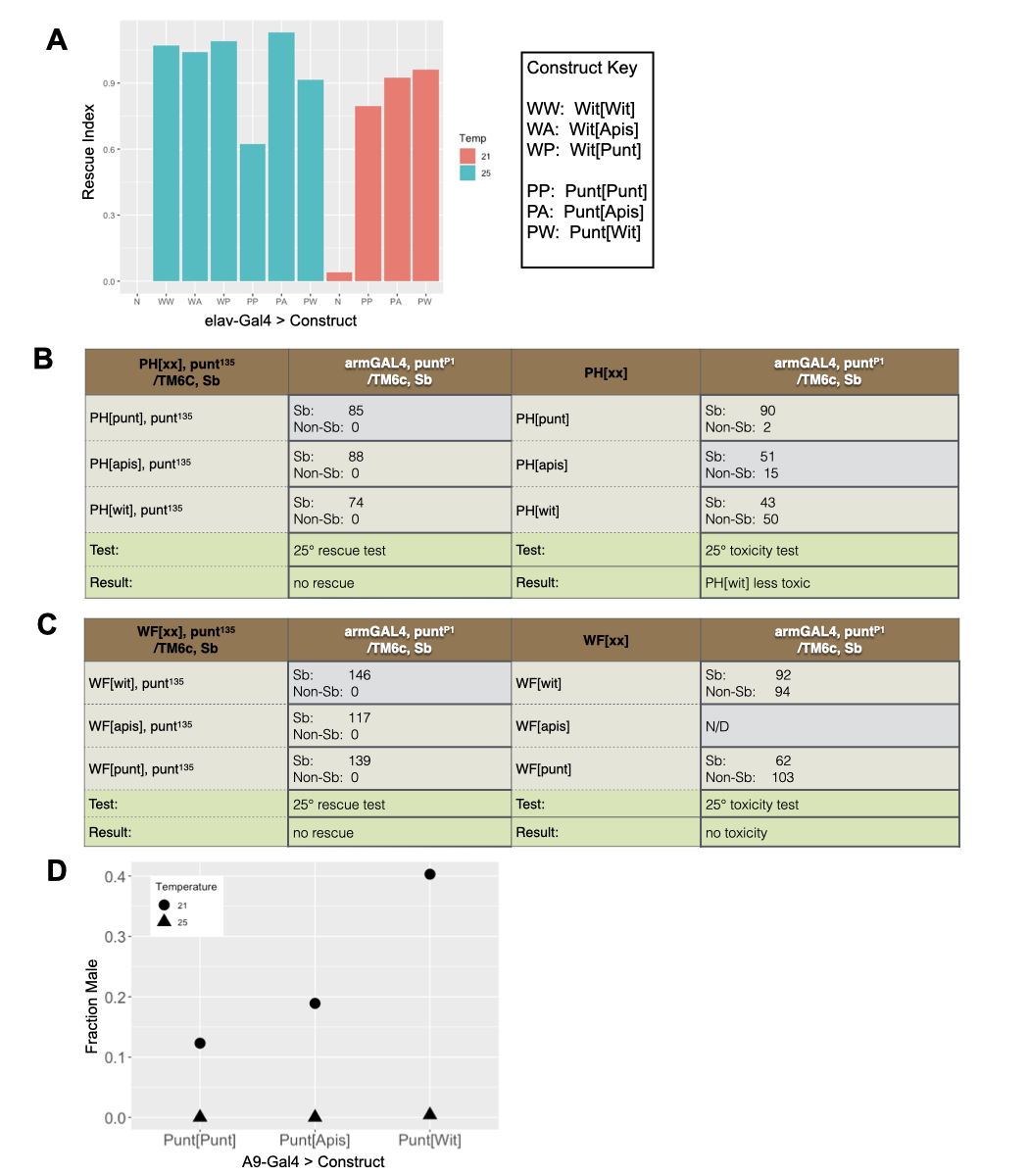

Supplement: S7 Fig — (A) wit mutants were rescued to viability by overexpression of Punt or Wit with elav-GAL4, regardless of which version of the juxtamembrane sequence was used (see key). (B) arm-GAL4 driving UAS-Punt proteins did not restore viability of put135/P1 mutants. PH[xx] indicates Punt-HA harboring the BLT sequence indicated in brackets. WF[xx] indicates Wit-Flag harboring the BLT sequence indicated in brackets. Toxicity tests in a put heterozygote background show significant lethality from ectopic expression of Punt[Punt] and Punt[Apis], but not Punt[Wit]. (C) UAS-Wit proteins also did not rescue put mutants, regardless of the BLT status. Toxicity tests showed that in this case arm>Wit did not lead to lethality. N/D: Not determined. (D) Punt[Wit] was less toxic than Punt[Punt], as shown by differential temperature sensitivity of male (more expression from A9-Gal4) and female (less expression) animals. The data underlying the graph shown in this figure can be found in S1 Data. BLT, basolateral targeting. (TIFF) [file pbio.3001660.s007.tiff]

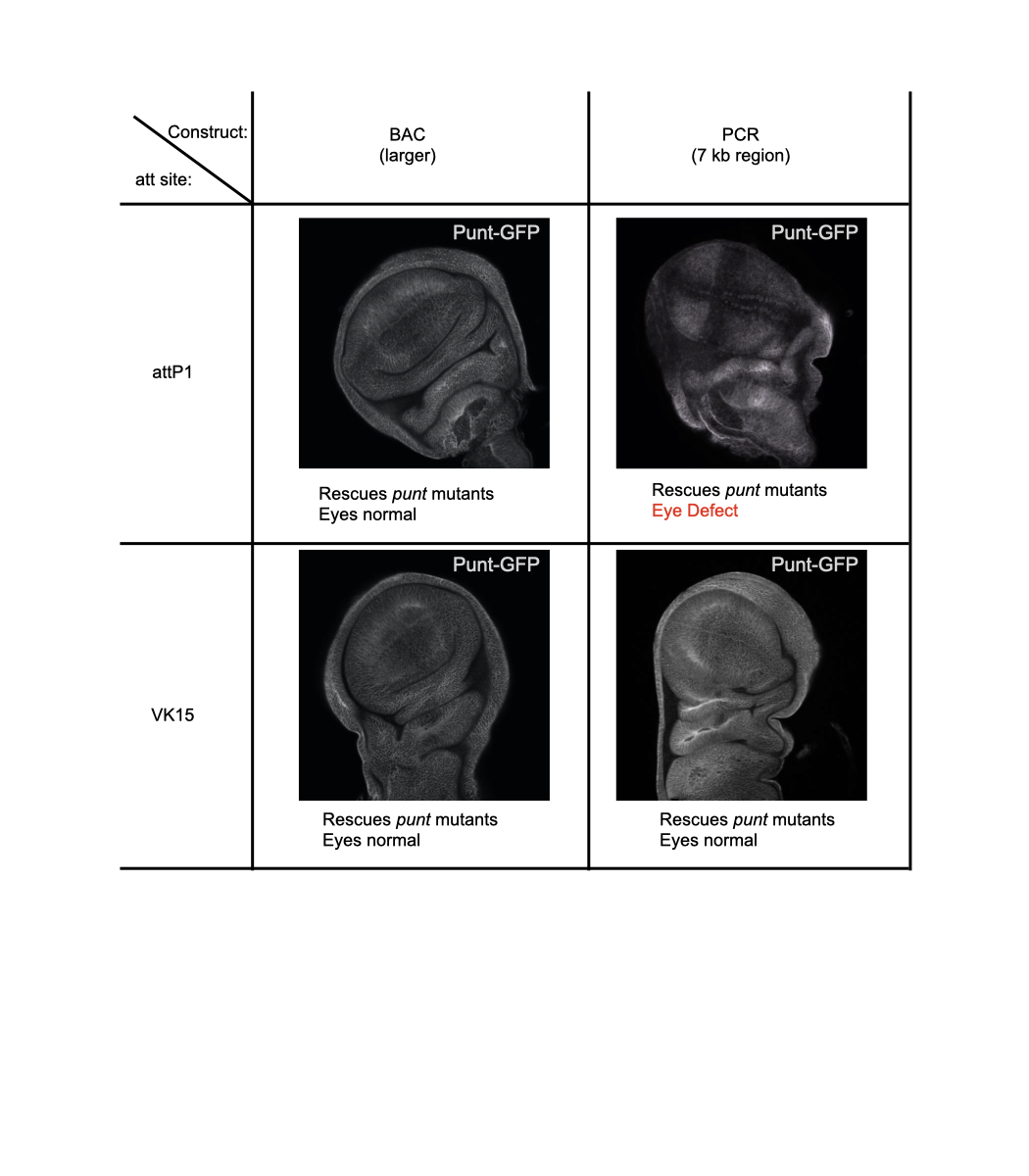

Supplement: S8 Fig — Patterned Punt-GFP detection was observed with the 7 kb rescue construct recombined into the attP1 docking site. However, a larger rescue construct at the same site showed uniform staining in the wing disc, as did the 7 kb construct at the VK15 docking site. There is thus a genome region influence only on the smaller construct. Variable eye defects (some adult eye tissue missing) were also only seen with the 7 kb construct at attP1, likely due to expression influences. (TIFF) [file pbio.3001660.s008.tiff]

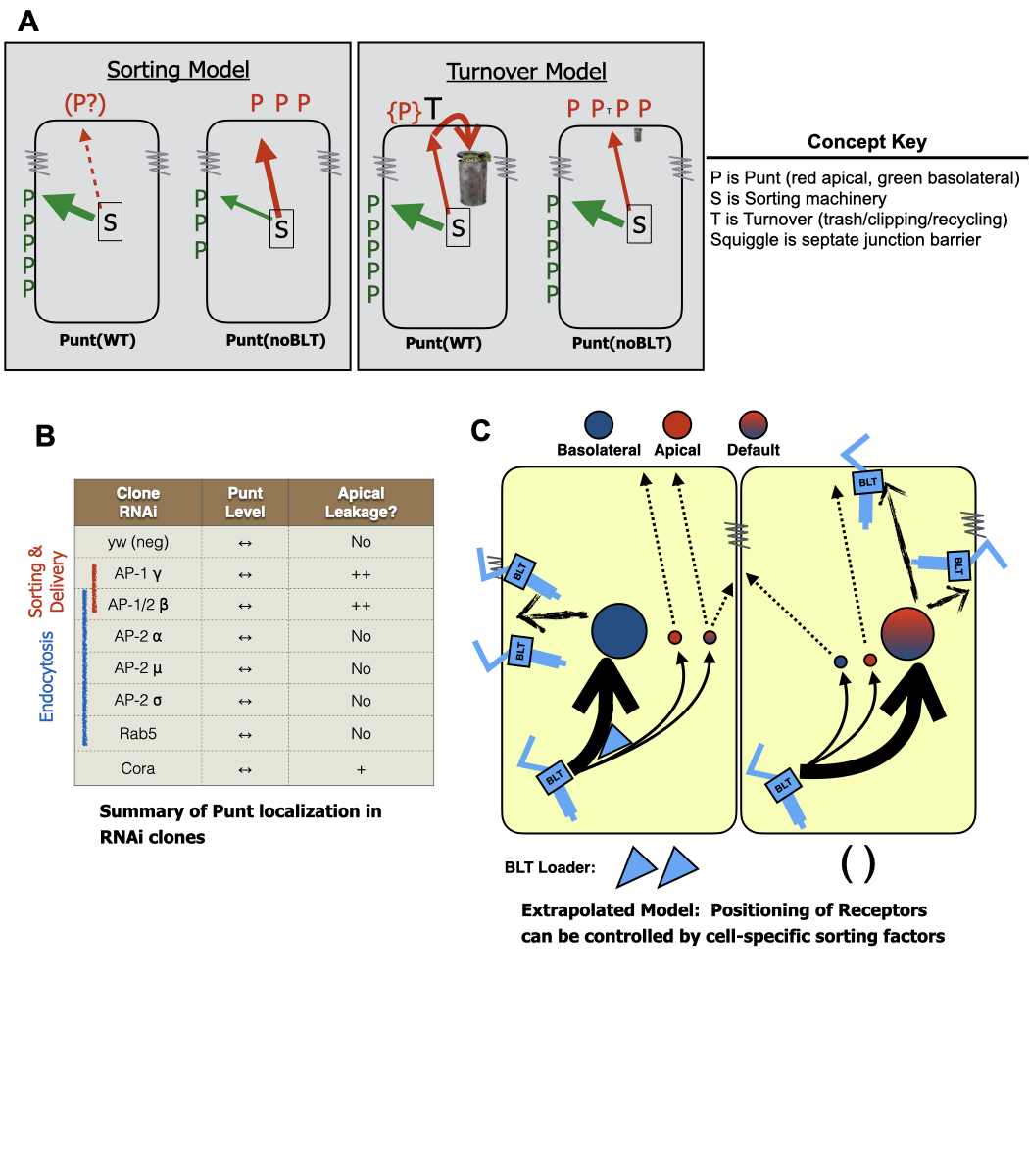

Supplement: S9 Fig — (A) Two general alternative mechanisms to achieve steady state basolateral restriction are presented, the Sorting Model and the Turnover Model. To differentiate between them, proteins required for sorting and delivery versus endocytosis were knocked down by RNAi to determine if localization of Punt-GFP was altered. Summarized results are shown in the table (B). (C) Based on results in Drosophila epithelia showing that the Punt BLT is a tissue-specific basolateral determinant, we propose a sorting paradigm utilizing tissue-specific loaders to direct receptors with targeting motifs to general or restricted membrane domains. BLT, basolateral targeting; RNAi, RNA interference. (TIFF) [file pbio.3001660.s009.tiff]
