# Supplementary material for: A juxtamembrane basolateral targeting motif regulates signaling through a TGF-β pathway receptor in Drosophila
Source: PLoS Biol. 2022 May 20;20(5):e3001660. doi: 10.1371/journal.pbio.3001660 (PMC9162340; doi:10.1371/journal.pbio.3001660)
Supplement: Attachment — Submitted filename: Response_to_Reviewers.docx [file pbio.3001660.s011.docx]

Response to Reviewers (* denotes author response)

We thank the reviewers for their comments and insights into our manuscript. The changes made in response to the questions and requests should make some key findings clearer to readers. We have also added new data that strengthens our main model that Punt is actively targeted to the basolateral membranes in the wing disc to support Dpp signaling.

Rev. 1:

In this manuscript, O'Connor and colleagues utilize the wing epithelium of Drosophila to analyze the membrane localization of Dpp receptors Tkv, Punt and Wit, to identify the evolutionary conserved relevant regions aimed at targeting these receptors to the apical or basolateral sides of the epithelium and to characterize the functional relevance of this targeting to Dpp signaling and patterning. The paper is well written, the main messages are subdivided into independent sections and figures, and the biological question is timely and relevant in the context of signaling. Authors focus on the relevance of the localization of Punt in particular, and identify a 19 aa-long region required for basolateral localization of the protein in the wing epithelium. I have two major concerns (one technical and one more profound):

(1) Basolateral localization of Punt is not convincing.

- Figure 1C: The basolateral localization of Punt-GFP is not convincing. Thus, "Overexpressed Punt protein exhibited basolateral restriction, with nearly all of the protein staying basal to the septate junction" is not supported by the data. Figure 1C: no labeling of FasIII was shown in this panel. I would suggest authors to perform cross-sections of the epithelium to support better evidence for the preferential localization of Punt (basolateral) and Wit (apical).

* This is a key piece of data, so we need readers to be on board with the type of data presented, and the basic observation that Punt is largely "below" the junctions. We have used FasIII and Dlg interchangeably, but grew to prefer Dlg because it gives a cleaner SJ "stripe" (e.g. Fig 1C). The cross-section reconstructions are another way to show the same result as the 2D profiles. The advantage of xz profiles is that it looks more like an archetypical single layer epithelium and is thus easier for many readers to comprehend. Some downsides are the compromised z-axis resolution, that it requires projections to reconstitute the image, and that the peripodial membrane sometimes enters the field. The circle-wrinkle view is a simple way to answer the same question, and has been deployed to nice effect by other labs (Bilder, Fehon, etc). But, it does require some explaining, which we attempted to do in Figure 1. The root cause of the situation is the wing disc being a 2D epithelium folded into a 3D structure.

- Figure 2F-G: authors should label apical and basal sides of the epithelial sheet.

* Agreed. Labels have been added to the Figure panels in addition to the Legend.

- Figure 3B-D: Again, authors should perform cross-sections of the epithelium to support better evidence for the preferential localization of wt Punt (basolateral) when compared to truncated versions of the protein. Data in Figure 3-S2 is much clearer where the profile of panel D'' is perhaps the most convincing to state the basolateral localization of wt Punt. The apical enrichment of Delta-10 in panel E'' is clear on one side but on the other. Authors should change it with more convincing data. The profile in panel F'' is very convincing. Why is the Delta-BLT and not the full cytoplasmic truncation localized apically in MDCK cells?

* xz projections were added for the Δ10 and Δ19 constructs, shown along with the 2D profile for comparison in an updated Figure 3.

* There is considerable variation from spot to spot in how the profiles look, and we are reluctant to select too strongly for the clearest ones. There is some signal from the staining techniques at the junctions, so we don't expect the signal for Punt to be zero. Agreed regarding E'', but this is in line with partial apicalization with Del10.

* Full cytoplasmic truncation is represented by the BA Shuttle in Figure S2. It is a little hard to see, but there is apical signal in addition to basolateral. A complete cytoplasmic truncation in the native Punt context was not tested. Rather we combined the chimera data with the C-terminal truncation data to predict that the Basolateral determinant is in the 19 aa juxtamembrane region, and this was born out by the unrestricted localization of Δ19 proteins in MDCK cells and wing epithelium.

- Figure 4: The profile of Punt-Apis should be shown to demonstrate its BL localization. Authors use the Punt-Wit quimeric protein and its apical enrichment to state that "basolateral targeting is a specific function of Punt juxtamembrane sequences". These data indicate that the cytoplasmic domain of Wit targets the protein to the apical side of the epithelium. Authors should make the Punt(Apis) lacking the cytoplasmic domain (or the BLT region) to experimentally demonstrate their statement. Data in panel 4B are shown fly Punt? If so, this should be stated as Punt (Drosophila). Profiles of panels I, J should be included.

* We were too terse with the descriptions of the various protein variants (e.g. large chimeras of Punt and Wit, C-terminal truncations, internal deletions, BLT-only swaps, point mutants). This generated some confusion about what the constructs are, and thus what their localization tells us. The Punt[Wit] protein is all Punt, except the native BLT is replaced by the equivalent region of Wit. Similarly, Punt[Apis] is all Drosophila Punt except for the BLT region, which is from Apis Punt. Thus, the partially apicalized location of the Punt[Wit] protein but the basolaterally restricted localization of Punt[Apis] indeed indicate that basolateral localization is an activity of Punt BLT sequences (at least for the two insect species we tested). We have added language to the Results and the relevant Legend to make the nature of these constructs more salient.

* Punt-Δ19 behaves like Punt[Wit]. We interpret this as evidence that the loss of the BLT, and not any activity of the Wit juxtamembrane segment, causes the change in membrane targeting. To buttress the point that Punt[Wit] is not changing behavior because of an active feature of the Wit juxtamembrane segment, we tested a Wit-Δ19 construct and found that it has similar apicobasal distribution as Wit (Updated Figure4K, with profiles for 4H-K shown in Figure S6).

* The issue of potential Wit apical targeting is interesting in its own right, and is related to how we describe the basolateral restriction of Punt. There is a visible apical stripe, but we have also noted that there is substantial basolateral protein detected. It isn't clear if the apical enrichment reflects some targeting, or if it is closer to a default distribution. It is striking that Punt without a functional BLT gets distributed like Wit. Multiple interpretations are possible, but we feel this is consistent with our larger model that Punt is actively targeted to the basolateral membrane domains. The issue of basolateral targeting vs apical exclusion is a common question we get about this project. It is tempting to dismiss this as a semantic choice. However, mechanistically they may be quite different. At times we do use the term apicalized (which may not be a proper word!) for brevity to indicate loss of basolateral restriction. Based on the totality of our results (both the deletion and substitution variants in this section, and the trafficking factor data later in the paper) we strongly favor the basolateral targeting model. In this view, the apical Punt observed when the BLT is disabled in cis (deletion) or in trans (loss of AP1 complex) reflects than in the absence of the normal polarized sorting pathway, Punt is still delivered to the plasma membrane, but in a generic apicobasal manner.

* Punt[Punt] is simply the control construct for the other BLT substitutions, and is named in parallel to the others (e.g. this is Fly Punt with its native BLT sequence).

* Profiles for the Punt[BLT] series, including Punt[Apis], are now included in S5.

- Figures 5E-J: Profiles of panels 5E-G required. It's sad to see authors do not use these constructs (instead of overexpression experiments) to present evidence of the requirement of these 19 aa to localize Punt basolaterally.

* We have now analyzed the membrane distribution profiles for the constructs used in the rescue experiments. To deal with the non-uniform expression pattern imposed by the landing site, we collected profiles from the same quadrant for multiple discs from the three constructs. In addition to showing representative profiles, we calculated an "Apical Index," which confirms that the EG point mutant has a mild loss of basolateral restriction and the Δ19 protein has more significant apicalization. The Apical Index for wild-type Punt is not zero because we did not attempt to subtract staining background, and because there is a small amount of apical protein detected with all the wildtype Punt constructs and detection methods we have used.

(2) Functional relevance of basolateral localization of Punt. Perhaps the most important data in this regard are shown in Figure 6F-M where a rescue construct of Punt lacking the BLT region (the one required to target Punt basolaterally) is able to almost completely rescue Dpp signaling and the adult wing phenotype. In this context, I wonder whether these data indicate that Punt localization is not relevant (which might frustrating and unexpected) or simply because the BLT region is not the one that targets Punt basolaterally but the one that excludes it from being localized apically. In order terms, all data can be explained by "ectopic" expression of Punt in the absence of BLT and not by loss of Punt basolaterally (where it binds the Dpp ligand. Thus, perhaps the major conclusion of these data is that ectopic localization of Punt in the apical domain is not relevant. I agree, though, that the BLT is required for animal fitness (as shown in Figure 5), but it's not demonstrated whether reduced fitness is a consequence of the protein being misplaced in the epithelium.

* The functional relevance of loss of Punt basolateral restriction is indeed key. Our final model is that Punt needs to be basolateral in the wing disc to support robust Dpp signaling. The lack of activity of apically-presented Punt was indeed a surprise to us, as mislocaliztion in two other systems can lead to ectopic signaling. Given a fixed pool of Punt, the impact of apical Punt is indirect: By diversion, it dilutes the Punt in the basolateral active zone. We have added two types of data to support this model: 1) AP1gamma clones have reduced basolateral Punt-GFP reporter (a "trans" test as it relies on altering the readout of the BLT) (Fig7K). 2) We repeated p-Mad staining in wing discs with 2 copies of the key rescue constructs. With increased dosage, the Δ19 construct supports Dpp signaling as well as the WT (Fig 6N-O). In this context, it make sense why there are relatively minor wing defects in Dpp-dependent patterning, since only a fraction of Punt is misdirected to the inactive zone. It is likely we have sensitized the system when testing one copy of Punt to rescue punt mutants. Partial reduction of one dose puts the wing in the "danger zone" where signaling is weak and patterning is not robust.

Minor issues: data presented in Figure 5A-C are out of context (from my point of view) and some of these data (eg- lethality) not quite conclusive. Labels of apical vs basal should be included in all the figures. Data presented in 7E-H not very convincing. Some of the last figures are difficult to interpret.

* To minimize disruption to the data flow, we have moved several data panels to the Supplement that deal with activity of over-expressed Punt and Wit protein variants. We retained data showing that the degree of ectopic activity of over-expressed Punt depends on the BLT. The lethality itself is clear, but we agree that the interpretation is not straightforward as it relates to mislocalization (see response to Rev 3 below). Combined with the later endogenous-level constructs, the lethality data in Figure 5 and later pMad staining, this supports the claim that an intact BLT is positively correlated with Punt signaling output.

* Apical and basal are now labeled in Figures where there is a defined axis. In 2D wing disc images, there isn't a fixed apicobasal axis as it varies with the contours of the epithelium. In these cases we indicated polarity with arrows/arrowheads (e.g. the updated Figure 7E-H) or relied on the relative orientation of SJ and apical markers.

* We have wrestled with the simplest way to show the apicalization of Punt in RNAi clones. Readers should be looking for patches of apical Punt in marked cells, but the 3D nature of the wing disc and the randomness of the clones complicates the view. For the revised Figure 7, we retain the same wing disc examples with some presentation changes: The main panel is cropped to better show the overall staining in and near the pouch, the Punt-GFP signal is in green to avoid potential Red-Fire-Green confusion, and the labels have been streamlined to mark two clone regions per inset with symbols indicating polarity and the presence or absence of ectopic apical Punt.

Rev. 2:

Using Drosophila as model system, the authors show the differential localization, i.e. basolateral versus apical, of two Dpp receptors, Punt and Wit. This observation provides the basis for their subsequent experiments that lead to the definition of the basolateral targeting (BLT) sequence in Punt, and of the role of this sequence, and of basolateral signaling, in Dpp's signaling activity in fly development.

Overall: I did not evaluate this manuscript from the perspective of a Drosophila developmental geneticist (not my area), but rather did so from my broad knowledge of TGF-b family signaling and biology.

This is a solid and straightforward story that is logically pursued, scientifically sound, clearly presented, and with the conclusions supported by the data. My comments relate to the presentation.

1. In the title and throughout the manuscript the authors talk about TGF-b receptors and their signaling. This is not appropriate, since Dpp is a homolog of BMP2 and BMP4. I realize that Drosphila labs and journals (editors) often use the term TGF-b when referring to Dpp, and that this comment may come over as annoying. However, considering Dpp as TGF-b is not only incorrect, but it also creates substantial confusion. For example, I have been in a thesis committee for a graduate student in a hybrid Drosphila/mouse lab, and the graduate student was pursuing TGF-b studies in mouse cell differentiation based on Drosophila observations related to Dpp, which was "advertised" as the homolog of TGF-b. This led to at least a year of wasted effort and a need to redesigning the thesis proposal with BMP (not TGF-b) being the homolog of Dpp. And of course BMPs often have effects that drastically differ from TGF-b.

* We are pleased that the contents of the story were well received. We also appreciate the reviewer's evident passion for TGF-β. We are certainly not trying to conflate Dpp with any of the proper TGF-β molecules encoded in deuterostome genomes. In other works, we have explicitly disambiguated the term "TGF-β" as it applies to Drosophila. Simply put, the main usage we intend here is to refer to the TGF-β signaling pathway. This is a common and useful shorthand in the broader field (e.g. the highly-regarded book titled "The TGF-β Family"). At a high level we want our Punt BLT story to be viewed in the context of TGF-β pathway signaling. Given the evolutionary kinship of TGF-β pathway proteins, we suggest that polarized presentation of receptors may be a common regulatory feature of various flavors of TGF-β pathways, whether they follow the canonical branch descriptions or utilize so-called cross-talk interactions.

We have made some changes to the text to minimize potential confusion about TGF-β receptors (Punt vs TβR's vs all receptors in the various pathways). There is still a balancing act between over-simplifying and over-describing, and it is not possible to simultaneously strike the right note for all readers. For example, our data in the wing disc focuses on Punt as a Dpp/BMP receptor, but it is also a receptor for Activin family ligands, so we introduce it as a Drosophila Type II receptor of the TGF-β signaling pathway. We have also added text in the Discussion about potential roles of the BLT in Activin branch signaling. (As an aside, I would wager that given the chance Punt could function as a TGF-β receptor!)

2. Please rename "superfamily" into "family". The TGF-b family is a family, not a superfamily. Again this comment that may be dismissed as annoying.

* We had used "superfamily" as an umbrella term for all variants of the TGF-β signaling pathway, which isn't a technically correct usage. As suggested, we have removed the word from the manuscript.

3. The Discussion often reads as a rehashing of the Results. No need to repeat what you already wrote in Results. Make it shorter and eliminate the retelling of the Results, while keeping the Discussion to a critical and insightful discussion of the Results. As it currently stands, the Discussion does not give me much new insight that I did not find in Results.

* This observation is pertinent for the first portion of the Discussion. We have significantly shortened this section to reduce retreading the Results, and to let the more interpretative statements later in the Discussion stand out.

Rev. 3:

TGF-b signaling is tightly controlled at multiple levels. Spatial coordination of TGF-b ligands and receptors is one of the critical regulations to elicit a proper signaling output. In this study, Peterson et al. observed a restricted basolateral membrane localization of the TGF-b type II receptor Punt in Drosophila wing disc epithelia. They reveal a novel basolateral targeting sequence (BLT) at the juxtamembrane position of Punt by comparing amino acid sequences between TGF-b receptors and by analyzing a series of deletion mutants. punt mutants lacking the BLT showed wing developmental defects, lethality, and female sterility. Based on these results, they concluded that proper basolateral localization of Punt is essential for optimal TGF-b signal transduction in Drosophila.

Major comments:

1. The results supporting the requirement of Punt's BLT in wing development are convincing. However, whether and how Punt mislocalization leads to sterility and lethality is unclear. Indeed, only wing disc epithelia show the restricted Punt localization. Thus, the BLT deletion may differentially influence Punt activity in other tissues. The authors should directly address this discrepancy.

* We had a disclaimer in the Discussion section touching on this issue, but we have now added statements to clarify what is and isn't shown by our experiments. The link between apicobasal localization and signaling is indeed only shown for the wing disc. We don't know *why* viability and fertility depend on the presence of BLT sequence. We note that these may or may not be due to mislocalization, and that the phenotypes may be due to Activin branch signaling defects. It is interesting that, in contrast to Dpp signaling in the wing, a second copy of the Δ19 rescue construct did not restore full viability or female fertility, suggesting the signaling perturbations are not simply due to limited Punt levels. Further studies are needed to explore the function of the BLT sequence in other tissues.

2. The wing folding is useful for visualizing apicobasal protein localization. However, readers with untrained eyes may have difficulty interpreting the images from the folding regions. Therefore, please also provide conventional crosssection images. In addition, it will be critical to examine endogenous receptor localizations since antibodies and transgenic lines to visualize their endogenous expression are available.

* This comment about folding neatly encapsulates the issue of imaging folds. It works (and in some respects is better; see above), but is difficult to explain succinctly to researchers who don't routinely image wing discs. We have added cross-section projection images for Figure 1 and Figure 3 to demonstrate the qualitative equivalence of the approaches for several key proteins.

* We have added several data points to address the behavior of endogenous (or endogenous level) proteins, with some technical limitations. For Punt, the antibody works well under over-expression conditions but the signal-to-noise for endogenous Punt is not acceptable. We thus show the basolateral preference of the Punt-GFP rescue construct (Figure 1). For Wit, we use the Fly-FOS construct stained for GFP to show the localization relative to the apical membrane (Figure 1). For Tkv, we use the antibody to detect endogenous protein and a YFP protein trap as a reporter (Figure S1).

Minor comments:

1. The authors stated that the Punt deletions (delta 10 and 19) did not destroy its signal transduction activity. However, as shown in Figure 3-figure supplement 1, the delta 19 mutant seems to have a dominant-negative effect.

* It is difficult to see everything going in the discs with excessive Punt because they are overgrown and distorted, but the Δ19-expressing sample does in fact have ectopic signaling as judged by widening of the disc (compare S3E to B; same xy scale despite different aspect ratio) and ectopic P-Mad in *vg*-positive regions (e.g. at the left side of the disc). Better description was added to Legend. It remains important to note that we don't compare relative amounts of ectopic activity between constructs because these are random P element insertions that don't have the same expression strength.

2. The authors used several transgenic lines and distinct detection methods to visualize Punt localizations but did not clearly describe how Punt proteins were detected in some figures.

* Receptor localization was determined by GFP fluorescence, or by IF using anti-Punt, anti-HA, anti-GFP, anti-Flag, or anti-Tkv antibodies. I believe these are now indicated in Figures as direct panel labels and/or in the Legend.

3. No data supports the conserved mutant protein's varied apicobasal localizations (page 13, line 21-23; Figure 4F).

* It was not stated explicitly, but the single profile shown was meant to show some apical localization on one side of the fold but not the other. The variability is a key part of the result. In parallel to the profiles, hopefully it is evident from the images that mutating the conserved residues leads to a distribution somewhere between wildtype and complete mutation/removal of the BLT region.

4. Please provide sample sizes for all quantifiable data.

* For numerical data, samples sizes are now indicated either directly on graphs or in the Legend. Underlying data for the graphs has also been compiled as a spreadsheet. Images of wing discs are representative of at least 5 tissues per experimental batch, with each experiment conducted independently at least twice.

5. Do not use abbreviations without explanation.

* SJ, IF, et al should be defined upon first usage. Please let us know if there are still undefined abbreviations in the manuscript.

6. A large BAC... (data not shown). > A large BAC... (Figure 5-supplement 2). (page 19, line 14)

* Thank you for catching this. The original "data not shown" meant the rescue test with that construct was done qualitatively rather than quantitatively, but we have corrected the Figure call-out. Note that names of the Supplemental Figures are now Figure S1, S2, etc.
