# Supplementary material for: A juxtamembrane basolateral targeting motif regulates signaling through a TGF-β pathway receptor in Drosophila
Source: PLoS Biol. 2022 May 20;20(5):e3001660. doi: 10.1371/journal.pbio.3001660 (PMC9162340; doi:10.1371/journal.pbio.3001660)
Supplement: Attachment — Submitted filename: BLT_v3_ResponseToReviwers.docx [file pbio.3001660.s012.docx]

Response to Reviewers, Round 2 for Peterson et al BLT Research Article

04/26/2022

Reviewer comments are pasted with author comments marked with *.

Reviewers' comments

Rev. 1:

Authors have addressed my concerns satisfactorily.

However, important issues that should be seriously addressed by the authors before considering it for publication include:

(1) Statistics and description of intensity profiles and pMAD signal intensity quantification are unfortunately lacking and should be included. Software used, details on statistics and a table summarizing all these data should be included.

(2) The lines where the profiles come from should be depicted in all figures.

(3) Position and size of labels and even font type and size appear to be rather chaotic.

(4) Scale bars for all panels should be provided

(5) Histograms and plots should be "aesthetically" improved.

*1. We have added information in the Methods section and updated the supporting Numerical_Data file. For pMad staining quantification shown in Fig 6E we have described the approach, and the raw data is included in the data file with a note indicating which data column was used to generate the boxplot. Intensity profile generation was described already in the Methods section. For the "Apical Index" shown for the revised Fig 5F, we have added more information to include the statistical test used (Tukey's multiple means testing carried out in R). For both analyses, we have added statistical information to the Numerical_Data file that will permit replication of our data handling. Between the graphs and the underlying data we propose that the quantitative data presentation should be sufficiently clear.

*2. Markers have been added to several panels in Figure 1. Note that to avoid obscuring the underlying image, we do not overlay the profile lines, rather employ delimiting arrowheads.

*3. Thank you for drawing attention to some of the varied presentation issues. We have attempted to harmonize the font, style, and position of Figure labels. Note that sequence alignments are intentionally based on a fixed-width font. Also, some of the text embedded in the images generated by FIJI or R may not match that added during Figure layout. Finally, in some cases the placement of labels was done to minimize obscuring underlying images, so they may not be rigidly consistent from figure to figure.

*4. Image panels now have scale bars, with the exception of xz and yz projections that accompany xy images in S1 Fig, where the scale is explicitly stated in the legend to apply to all three views.

*5. We used several graph styles in the main Figures. For "counting data" we used grouped bar graphs (Fig5G, Fig5H, and Fig5I) or a scatter plot (Fig5A). For quantitative data, we show box plots (Fig5F and Fig6E). The groupings, symbols, and colors are intended to highlight relationships in the data (e.g. the same genotype at different temperatures in Fig 5A, or different copy number of the same rescue construct in Fig5G).

The aesthetics are largely "standard" outputs for the common ggplot2 package for R, and we admittedly did not expend much artistic effort to polish the appearance. The primary purpose of the graphs is to visually display and bring out relationships in the data. If there are specific informational elements that are unclear we will gladly revise the graphs. However, given the subjective nature of aesthetics, we are unable to divine the steps required to efficiently satisfy this request.

Rev. 2:

This is a nice and solid paper, worth publishing and reading.

Minor: page 20, line 22: "add" should read "adds".

* Thank you for the encouraging words, and for reading the discussion section carefully. The noted grammar mistake has been fixed.
